# VOR gain of lateral semicircular canal using video head impulse test in acute unilateral vestibular hypofunction: A systematic review

**DOI:** 10.3389/fneur.2022.948462

**Published:** 2022-12-08

**Authors:** Mohamad Alfarghal, Mohammed Abdullah Algarni, Sujeet Kumar Sinha, Aishwarya Nagarajan

**Affiliations:** ^1^Otorhinolaryngology - Head and Neck Section, Surgery Department, King Abdulaziz Medical City, Jeddah, Saudi Arabia; ^2^Otorhinolaryngology - Head and Neck Section, Surgery Department, King Abdulaziz Medical City, King Saud Bin Abdulaziz University for Health Sciences, Jeddah, Saudi Arabia; ^3^Department of Audiology, All India Institute of Speech and Hearing, Mysore, India

**Keywords:** VOR gain, vestibular hypofunction, vHIT, AICA, PICA

## Abstract

**Introduction:**

Acute unilateral vestibular hypofunction is characterized by sudden onset of vertigo or dizziness, vomiting/nausea, gait instability, and nystagmus. This is commonly described as an acute vestibular syndrome and usually attributed to vestibular neuritis; however, up to 25% of acute vestibular syndrome is caused by a stroke of posterior circulations. The video head impulse test is a recent tool in the vestibular test battery that assesses the vestibule-ocular reflex by measuring the VOR gain and recording overt and covert saccades, these findings have been found to be helpful in the diagnosis of various vestibular disorders.

**Method:**

A literature search was conducted in databases, including PubMed Central, PubMed, and Web of Science. All the articles that define video head impulse test (vHIT), acute vestibular hypofunction, and vestibular neuritis were considered for the preliminary search. No limits were placed on the date of publication. The searches were limited to studies with full-text availability, published in English, and including human subjects. Search words such as “head impulse test,” “video head impulse test,” “vestibular ocular reflex,” “acute vestibular syndrome,” “acute vestibular hypofunction,” “vestibular neuritis,” and “vHIT in central vestibular disorders” were entered into different databases in different combinations using boolean operators such as AND, OR, and NOT.

**Results:**

Searches across different databases, including Web of Science, PubMed Central, and PubMed, resulted in a total of 1,790 articles. Title screening was done for all the articles. Out of the 1,790 articles, we found that 245 articles were related to vestibular hypofunction i.e., 1,545 articles were removed at this stage. A further 56 duplicate articles were removed. This led to a final screening of 189 articles. The exclusion criteria included unavailability of full text, studies reported in languages other than English, case reports, reviews, and articles including participants having other comorbid conditions. This final screening led to 133 articles being excluded, which led to the full-text screening of 56 articles. After screening the full-text articles as per the eligibility criteria, 21 articles were found to be eligible for the systematic review. Among the remaining studies, six articles were excluded due to different specific reasons. A total of 15 articles were included in this systematic review. The mean VOR gain for the patients with vestibular neuritis was 0.48 ± 0.14 for the ipsilesional ear, whereas the mean VOR gain was > 0.80 in the contralesional ear for all the patients with acute vestibular neuritis. In patients with PICA lesions, the VOR gain for the ipsilesional ear was 0.90 (range 0.87–0.94) and for the contralesional ear was 0.88 (range 0.84–0.93). In patients with AICA lesions, the mean VOR gain was variable. Based on the above mean VOR gain findings, the authors propose the following adjective description scale of VOR of the lateral canal using vHIT: normal VOR gain above 0.80, mild VOR gain loss for 0.70–0.79, moderate loss for 0.69–0.4, severe loss for 0.39–0.2, and profound loss for < 0.2.

## Introduction

Every year, approximately 15 to 20% of adults suffer from dizziness, wherein about a quarter is accounted for due to vestibular dysfunction, which has an annual incidence and prevalence of 1.4 and 5%, respectively (1). One of the major reasons for prolonged vertigo which patients encounter is acute unilateral vestibular hypofunction and its mimics. Acute unilateral vestibular hypofunction typically presents as a sudden or acute onset of vertigo or dizziness, nausea/vomiting, gait instability, and nystagmus, a constellation of manifestations called acute vestibular syndrome. The condition is usually attributed to vestibular neuritis; however, up to 25% of acute vestibular syndrome cases are caused by posterior fossa stroke (2–4).

Vestibular neuritis is the most common cause of acute vestibular syndrome (2, 5). It selectively affects the function of the vestibular system and usually, the hearing is preserved. In the acute stage of the disease, is quite challenging to differentiate vestibular neuritis from posterior circulation stroke; clinical signs such as (a) direction of the fast phase of spontaneous nystagmus, (b) ocular torsion, (c) skew deviation, and (d) postural instability can help clinicians differentiate between the two conditions (6).

Anterior inferior cerebellar artery (AICA) territory infarcts comprising 1% of ischemic cerebellar strokes are less common than posterior inferior cerebellar artery (PICA) infarcts (7, 8). Around 10% of patients with cerebellar infarction do not show clinical neurological manifestations apart from vertigo or dizziness (9). Sometimes, isolated central acute vestibular syndrome is referred to as *pseudoneuritis* since it closely mimics the signs of vestibular neuritis (10).

One of the recent vestibular function assessment tools is the video head impulse test (vHIT). vHIT is used to assess vestibular functions by comparing head velocity to eye velocity where brief, unpredictable head impulses are given. vHIT measures the functions of all six semicircular canals individually and their corresponding nerves (superior and inferior vestibular nerves) when the head impulses are provided in horizontal, RALP (right anterior and left posterior), and LARP (left anterior and right posterior) planes. It assesses the vestibulo-ocular reflex (VOR) and records the presence of overt and covert saccades, thus aiding in the differential diagnosis of various vestibular disorders. VOR is the physiological mechanism of the vestibular system that generates equal and opposite eye movements when there is a head turn, which helps us to have clear vision during head movements.

The main measures in vHIT are the VOR gain, VOR gain asymmetry, and the presence or absence of compensatory saccades. The VOR gain assesses the function of the semicircular canals and their corresponding nerves on both sides. A clinical head impulse test and, afterward, a vHIT are beneficial in the early detection of isolated central acute vestibular syndrome, where traditional neurological signs are absent. vHIT has specific patterns in some neurological disorders such as Thiamine deficiency (Wernicke's encephalopathy) and multiple sclerosis with subclinical internuclear ophthalmoplegia. Generally, vHIT is a promising tool in differentiating the central and peripheral causes of acute vestibular syndrome (1, 11–16).

The acute stage is defined in this manuscript as within 1 week after the onset of symptoms. The acute phase of unilateral vestibular hypofunction points to the first few days, especially the first 3 days after the onset of symptoms (6). A 1-week cut-off permits the inclusion of more relevant studies to the review and, at the same time, avoids the inclusion of cases where recovery or vestibular compensation might significantly change the results of VOR gain. Patients with unilateral vestibular neuritis show significantly reduced VOR gain values on the ipsilesional side with normal VOR gain in the opposite ear. Patients with PICA infarcts demonstrate normal or near-normal VOR gain on both sides, whereas patients with AICA infarcts have a heterogeneous pattern of VOR gain values (17). There are also studies reporting bilaterally reduced VOR gain values in cases of vestibular neuritis (18).

Thus, using vHIT, we can differentiate between vestibular neuritis and a PICA infarct. However, in the case of an AICA infarct, we need to seek other clinical tests such as (a) gaze-evoked nystagmus, (b) a test of skew deviation, (c) head-shaking test, and (d) a test of hearing sensitivity. We cannot rely on vHIT findings alone to avoid misdiagnosis (2, 19–24). One study reported that the horizontal head impulse test in the HINTS test battery has a higher sensitivity (~91%) and specificity (~100%) when compared to MRI-DWI in the diagnosis of acute vestibular syndrome due to posterior circulation stroke in the first 48 h (22).

The main aim of this research is to systematically review various patterns of VOR gain values in acute unilateral vestibular hypofunction and its mimics using vHIT across research studies. This study aims to address the possibility of differentiating vestibular neuritis from PICA and AICA infarcts based on ipsi- and contralesional VOR gain values. Such a categorization of peripheral and central causes of acute vestibular Syndrome using VOR gain values obtained through vHIT would be important for clinicians, especially those attending acute vertigo. Another aim of this study is to present a grading scale for VOR gain values to facilitate interdisciplinary communication and to be used in clinical practice to help in the differentiation between vestibular neuritis and posterior circulation infarct and in the diagnosis of other vestibular disorders (10). The descriptive categorization of VOR gain proposed in this research is similar to Goodman's scale for hearing impairment (25). Adjective descriptors of hearing loss levels are still the preferred classification among clinicians, and possibly the proposed adjective description of vestibular VOR gain loss will be perceived similarly by clinicians (26).

## Methods

### Searches

A literature search was conducted in databases, including PubMed, PubMed Central, and Web of Science. The review was conducted in accordance with preferred reporting items for systematic reviews and meta-analysis (PRISMA). All the articles that defined vHIT and acute unilateral vestibular hypofunction were considered for the preliminary search. No limits were placed on the date of publication. The searches were limited to studies with full-text availability, that were published in English, and had human subjects. Search words such as “video head impulse test and acute vestibular syndrome,” “acute vestibular Hypofunction and video head impulse test,” “vHIT in central vestibular disorders,” “vHIT and vestibular neuritis,” “vHIT and vestibular Hypofunction,” “vHIT and AICA stroke,” and “vHIT and PICA stroke” were entered into different databases in different combinations using boolean operators AND, OR, and NOT. Study designs such as retrospective and prospective observational studies, cross-sectional studies, longitudinal studies, and randomized clinical trials were included. Studies that do not report direct or indirect observations or original data, case studies, letters to editors, expert opinions, animal studies, conference abstracts, and reviews were excluded from the present study.

### Condition or domain being studied

1. Evaluation of lateral semi-circular canal function in cases with acute unilateral vestibular hypofunction and its mimics.
2. Ipsilesional and contralesional VOR gain value of the lateral canal in cases with acute unilateral vestibular hypofunction and its mimics.

### Participants/population

**Inclusion:** Individuals of any age presenting with signs and symptoms of acute unilateral vestibular hypofunction were considered for the study. Studies also included normal individuals without acute vestibular hypofunction as control groups.

## Analysis

### Data extraction (selection and coding)

The titles and abstracts of all the obtained articles from different databases were screened by two authors (AM, SKS) independently. Only the articles fulfilling the inclusion criteria were included. The reference list of the included studies was further reviewed to obtain additional relevant articles. Any discrepancies or disagreements regarding the methodology of the article were resolved through discussions between the two authors. A full-text screening of the included studies was done in the second stage of analysis through the same process. The reasons for exclusion were documented and reported at this phase following the Preferred Reporting Items for Systematic Reviews and Meta-Analyses (PRISMA) standards (27). The risk of bias was calculated, and the two reviewers carried out the assessment for the same independently. Abnormal or normal VOR gain, ipsilesional and contralesional VOR gain value of the lateral canal, and the presence or absence of compensatory saccades were taken as the data elements of interest.

### Risk of bias analysis

The Quality Assessment of Diagnostic Accuracy Studies (QUADAS-2), an evidence-based quality assessment tool, was used to assess the risk of bias in the included studies by the two independent authors. QUADAS-2 has been specifically developed to be used in systematic reviews of diagnostic accuracy studies, with four critical domains, including patient selection, index test(s), reference standard, flow, and timing. After obtaining the rating for each question, the percentage of “yes” was calculated for each study by finding the total number of “yes” out of 11 questions. Any disagreements regarding the quality assessment of the article were resolved through discussions between the two authors.

### Strategy for data synthesis

The data synthesis was carried out by analyzing the homogeneity of the data, and different analysis parameters such as VOR gain (ipsilateral and contralateral).

## Results

**Figure 1:**
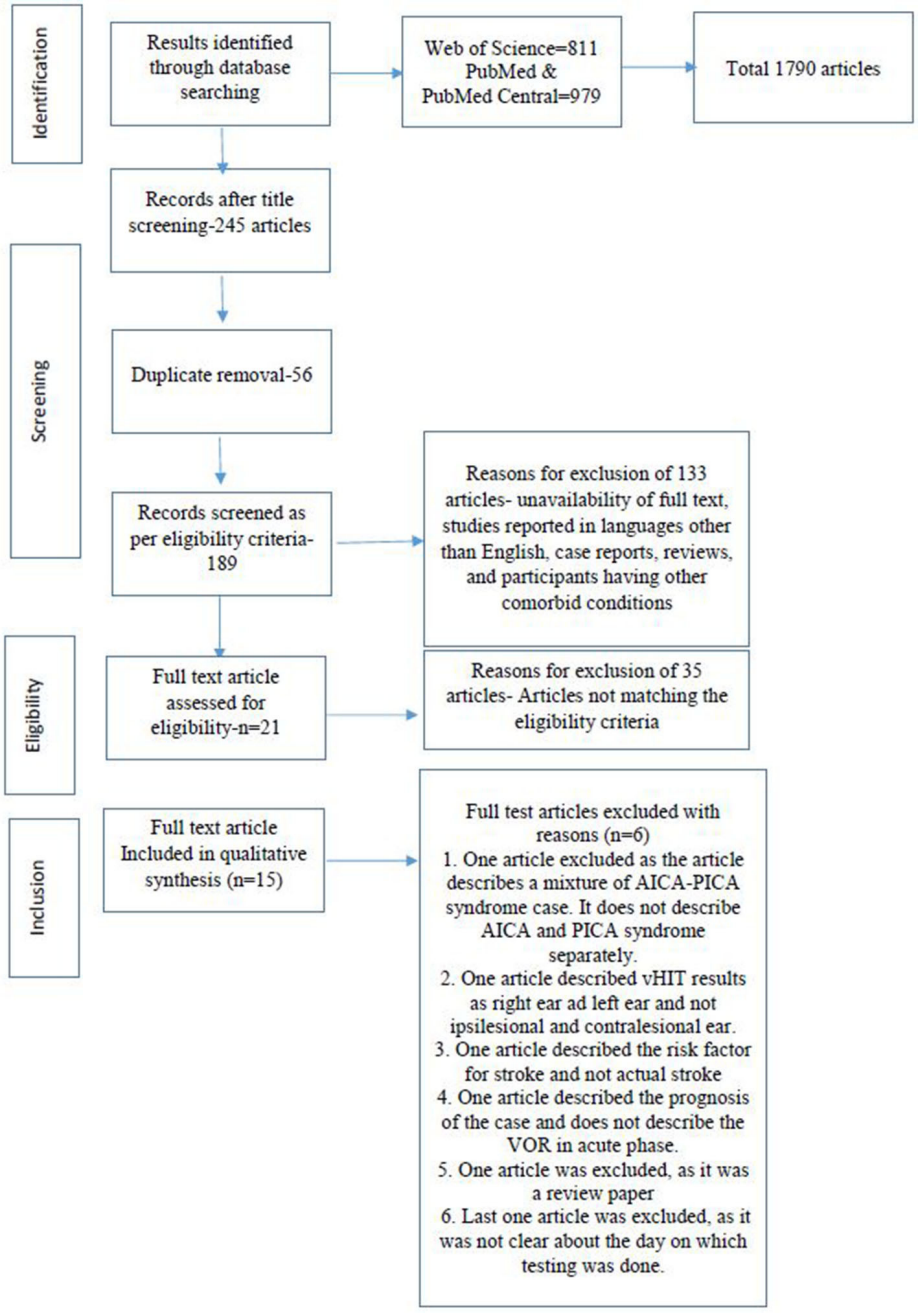
PRISMA chart for systematic reviews and meta-analysis.

Searches across different databases, including Web of Science, Pubmed Central, and PubMed, resulted in a total of 1,790 articles. Title screening was done for all the articles. Out of the 1,790 articles, we found 245 articles that were related to vestibular hypofunction meeting the objectives of the study i.e., 1,545 articles were removed at this stage. Again, 56 duplicate articles were removed. This led to a final screening of 189 articles. Using the exclusion criteria, which included the unavailability of full text, studies reported in languages other than English, case reports, reviews, and participants with other comorbid conditions, 133 articles were excluded, which led to the full-text screening of 56 articles. After screening for full text, as per the eligibility criteria, 21 articles were found to be eligible for the systematic review. Among the remaining 21 studies, six articles were excluded due to the following reasons. One article was excluded as the article describes a case of a mixture of AICA-PICA syndrome and did not describe the AICA and PICA syndromes separately. One article described the vHIT results as right and left ear and not ipsilesional and contralesional. One article described the vascular risk factor for stroke and not an actual stroke. One article described the prognosis of the cases and does not describe the VOR in the acute phase. One article was excluded as it was a review paper, and the last article was excluded as it was not clear which day the testing was done (28–33). In total, 15 articles were included in this systematic review. The screening process and the reasons for exclusion are depicted in the PRISMA flow (Figure 1).

### Characteristics of selected studies

All the records included in this study analyzed VOR gain in cases of acute unilateral vestibular hypofunction and its mimics. The number of participants with acute unilateral vestibular hypofunction cases varied between 1 and 63 in the included 15 studies. VOR gain and saccades were analyzed in all the studies. The characteristics of the studies including participants and the findings of vHIT described in acute unilateral vestibular hypofunction cases and its mimics are given in Table 1.

**Table 1:** Characteristics of studies included in the review.

**S. No**	**Study**	**Equipment used**	**Number of patients**	**VOR gain of ipsilesional lateral canal**	**VOR gain of contralesional lateral canal**
1.	Nam et al. (34)	SLMED, Seoul, Korea)	17 patients with PICA	Mean VOR gain = 0.87 ± 0.13	Mean VOR gain = 0.84 ± 0.13
			17 patients with VN	Mean VOR gain = 0.56 ± 0.16	Mean VOR gain = 0.91 ± 0.04
2.	Blödow et al. (35)	Interacosutics.Middelfart, Denmark	52 patients with VN	Mean VOR gain = 0.43 ± 0.20	
3.	Halmagyi and Curthoys (36)	GN Otometrics, Taastrup, Copenhagen, Denmark	1 patient with VN	Mean VOR gain = 0.32 ± 0.09	Mean VOR gain = 0.86 ± 0.04
4.	Yang et al. (37)	GN Otometrics, Taastrup, Copenhagen, Denmark	63 patients with VN	Mean VOR gain = 0.49 ± 0.03	Mean VOR gain = 0.95 ± 0.12
5.	Manzari et al. (6)	GN Otometrics, Taastrup, Copenhagen, Denmark	28 patients with VN	Mean VOR gain = 0.39 ± 0.17	Mean VOR gain = 0.91 ± 0.11
6.	McGarvie et al. (38)	GN Otometrics, Taastrup, Copenhagen, Denmark	1 patient with VN	Mean VOR gain = 0.33 ± 0.05	Mean VOR gain = 0.64 ± 0.3
7.	Lee et al. (39)	(SLMED, Seoul, Korea)	13 patients with VN	Mean VOR gain = 0.58 ± 0.21	
8.	Mantokoudis et al. (17)	GN Otometrics, Taastrup, Copenhagen, Denmark	16 patients with VN 7 patients with PICA 3 patients with AICA	Mean VOR gain = 0.52 ± 0.04 Mean VOR gain = 0.94 ± 0.04 Mean VOR gain = 0.84 ± 0.10	Mean VOR gain = 0.87 ± 0.04 Mean VOR gain = 0.93 ± 0.04 MeanVOR gain = 0.74 ± 0.10
9.	Yoo et al. (40)	SLMED, Seoul, Korea)	23 patients with VN	Mean VOR gain = 0.55 ± 0.29	Mean VOR gain = 1.00 ± 0.17
10.	Kim et al. (41)	GN Otometrics, Taastrup, Copenhagen, Denmark	30 patients with VN	High velocity Mean VOR gain = 0.50 ± 0.26 Low velocity Mean VOR gain = 0.62 ± 0.25	
11.	Walther and Blödow (42)	Interacosutics, Middelfart, Denmark	20 patients with VN	Mean VOR gain = 0.41 ± 0.17	Mean VOR gain = 0.93 ± 0.08
12.	Redondo-Martínez et al. (43)	Otometrics, Taastrup, Copenhagen, Denmark	20 patients with VN	Mean VOR gain = 0.50	Mean VOR gain = 0.89
13.	Skorić et al. (44)	Interacosutics,Middelfart, Denmark	31 patients with VN	R Mean VOR gain = 0.61 ± 0.30 L Mean VOR gain = 0.63 ± 0.34	
14.	Chang and Schubert (45)	GN Otometrics, Taastrup, Copenhagen, Denmark	4 patients with VN	Mean VOR gain- = 0.57	
15.	Roh et al. (46)	GN Otometrics, Taastrup, Copenhagen, Denmark	21 patients with VN	Mean VOR gain = 0.52 ± 0.17	Mean VOR gain = 1.00 ± 0.13

VN, Vestibular neuritis; AICA, Anterior inferior cerebellar artery; PICA, Posterior inferior cerebellar artery; SD, standard deviation; V-OR, Vestibulo-Ocular reflex gain.

### Risk of bias analysis

The risk of bias analysis of all the studies showed a low risk of bias. Hence, all the studies were included in the final analysis and review.

### Analysis parameters

The most studied video head impulse test parameter is the VOR gain, calculated by instantly comparing the eye movement to the head movement, which normally should be the same amount of movement but in opposite direction. The exact method of calculating the VOR gain is different from one system to another. The ratio of the area under the eye velocity curve to the area under the head velocity curve is used in the ICS GN Otometrics system and SLMED system, but instantaneous gain is used in the Eyeseecam Interacoustics system (11, 34, 35). Both the ICS GN Otometrics and Interacoustics systems were validated against a magnetic search coil (11, 47). The SLMED system was validated against special calibration system using an artificial eye (Personal communication).

All the studies included in this systematic review process evaluated the VOR gain but the cut-off criteria between normal and abnormal VOR gain varied from study to study.

#### Vestibulo-ocular reflex gain in acute vestibular neuritis

Nam et al. (34) described the VOR gain values of the horizontal, posterior, and anterior semi-circular canals using the video head impulse test in 17 patients with vestibular neuritis. All the participants suffered acute prolonged vertigo, nausea or vomiting, and gait disturbance. The subjects were tested with vHIT in the first week after symptom onset. The diagnosis of vestibular neuritis was made based on a history of acute onset vertigo with the presence of unidirectional spontaneous nystagmus, abnormal clinical HIT and canal paresis, and the absence of central signs. The VOR gain was abnormal in all 17 participants for the horizontal canal. The mean ipsilesional VOR gain varied between 0.44 and 0.61 for all the participants.

Blodow et al. (35) evaluated VOR gain using the vHIT in 52 patients with unilateral vestibular neuritis. The diagnosis of vestibular neuritis was based upon the presence of rotatory vertigo, horizontal-rotatory spontaneous nystagmus, gait imbalance and falling, vomiting, nausea, and a pathological side difference (>25%) at bithermal caloric irrigation. The normal VOR gain threshold was set to 0.79. The presence of abnormal VOR gain was recorded in 94.2% of the patients with vestibular neuritis. The mean ipsilesional VOR gain value for patients with vestibular neuritis was 0.43 ± 0.20.

Yang et al. (37) recorded vHIT in 63 patients with acute unilateral vestibular neuritis. All the patients were tested within 1 week of the onset of vestibular neuritis. The diagnosis of VN was made based on a history of acute onset of severe prolonged vertigo lasting 24 h, the presence of spontaneous horizontal unidirectional nystagmus without hearing loss or middle ear pathology on clinical examination, and abnormal caloric test results. The VOR gain was affected in 87% of the patients with vestibular neuritis. The VOR gain of the lesioned side in patients with vestibular neuritis varied between 0.15 and 1.18 (mean ipsilesional VOR gain = 0.49 ± 0.03. The VOR gain for the contralateral side ranged between 0.70 and 1.28.

Manzari et al. (6) recorded vHIT in 28 patients with vestibular neuritis. All the participants in the study were tested within 6 weeks of the onset of vestibular neuritis. The diagnosis of vestibular neuritis was made based on a history of acute onset of severe, prolonged rotatory vertigo, nausea, postural imbalance, presence of horizontal nystagmus, and abnormal VEMP test results. Manzari et al. (6) further subdivided the patients with vestibular neuritis into two groups. The first group included the patients assessed within the first 72 h after the onset of symptoms and the second group included patients tested after 72 h. The mean ipsilesional VOR gain for 15 patients who were tested within 72 h was 0.39 ± 0.17. Lee et al. (32) reported a VOR gain in 13 patients with acute vestibular neuritis. The mean VOR gain for patients with acute vestibular neuritis was 0.58 ± 0.21.

Yoo et al. (40) reported a VOR gain in 23 patients with vestibular neuritis who were tested within one week after the onset of vertigo. The mean ipsilesional VOR gain was 0.55 ± 0.29 for patients with vestibular neuritis. Kim et al. (41) reported a VOR gain in 30 patients with vestibular neuritis. The VOR gain was measured at two different peak head velocities. The VOR gain in patients with vestibular neuritis was 0.50 ± 0.26 for high head velocity and 0.62 ± 0.25 for low head velocity. Walther et al. (42) reported a VOR gain in twenty patients with acute vestibular neuritis and the VOR gain was 0.41 ± 0.17 on the affected side and 0.93 ± 0.08 on the healthy side. Redondo-Martinez et al. (43) reported the VOR gain to be 0.50 in the lesioned ear of the participants with vestibular neuritis. Halmagyi and Curthoys (30) reported the VOR gain to be 0.32 ± 0.09 in the ipsilesional ear of one patient with vestibular neuritis. McGarvie et al. (38) reported the VOR gain to be 0.33 ± 0.05 in a single patient with vestibular neuritis.

To summarize, the VOR gain is significantly reduced in patients with acute unilateral vestibular hypofunction due to vestibular neuritis, when they are tested within 7 days of the onset of symptoms.

#### VOR gain in AICA and PICA lesions

Nam et al. (34) characterized VOR gain using vHIT in 17 patients with a PICA stroke. The study participants were included based on the presence of acute continuous vertigo, nausea or vomiting, and gait disturbance, seen within 7 days after the onset of symptoms. Patients with a previous history of stroke or vestibular disorders were excluded from the study. The diagnosis of a PICA stroke was confirmed with an MRI scan. The VOR gain for the horizontal canal in the PICA group was 0.87 ± 0.13 (range = 0.80 to 0.93). However, in 9 out of 17 participants the VOR gain was slightly reduced on both sides. The authors suggested that a VOR gain higher than 0.71 ipsilesional in acute vestibular syndrome can predict a PICA stroke, yet they recommended that this value should be validated in clinical practice.

Mantokoudis et al. (17) described the VOR gain in 7 patients with PICA and 3 patients with AICA stroke. The patients presented with acute vestibular syndrome. Patients with known prior vestibular oculomotor diseases, acute drug/alcohol intoxication, or new head trauma were excluded from the study. The VOR gain for individuals with PICA stroke was normal on both sides without VOR asymmetry. There was also no difference between the ipsilesional and contralesional VOR gain values. The VOR gain for the ipsilesional side was 0.94 ± 0.04 and the contralesional side was 0.93 ± 0.04. Three patients with AICA stroke had variable VOR gain values. The mean VOR gain value for individuals with AICA stroke was 0.84. Also, the mean VOR gain values on the contralesional side for the individuals with AICA stroke (0.74) were less compared to the ipsilateral side (0.84). Based on these findings, they concluded that if the normal VOR gain criterion was kept at 0.7 or more for suspected stroke, it could lead to the diagnostic accuracy of 90% for individuals with PICA stroke.

#### Mean VOR gain in vestibular neuritis & AICA and PICA lesion

The VOR gain for the patients with vestibular neuritis varied between 0.15 and 0.89 for the ipsilesional ear, whereas the VOR gain was < 0.80 in the contralesional ear for all the patients with acute vestibular neuritis. The mean VOR gain was calculated for the mean gain values reported in all the studies. The mean ipsilesional lateral canal VOR gain of all the studies is 0.48. In the contralesional ear of patients with vestibular neuritis, the mean VOR gain is 0.85 (range 0.50 to 1.00). In patients with PICA lesions, the VOR gain for the ipsilesional ear is 0.90 (range 0.87–0.94) and for the contralesional ear is 0.88 (range 0.84–0.93). In patients with AICA lesions, the mean VOR gain is 0.84 for the ipsilesional ear and 0.74 for the contralesional ear. Based on the above mean VOR gain findings, we propose the following scale for VOR gain calculations (Table 2).

**Table 2:** VOR gain loss grading scale.

**VOR gain**	**Grade**
≥ 0.8	Normal VOR gain
0.79–0.70	Mild VOR gain loss
0.69–0.4	Moderate VOR gain loss
0.39–0.2	Severe VOR gain loss
<0.2	Profound VOR gain loss

The proposed grading scale of the severity of VOR gain loss in acute unilateral vestibular hypofunction might help in understanding the etiology, predicting the outcome, and providing easier inter-professional and clinician-patient communication.

## Discussion

Different synonyms such as vestibular neuritis, vestibular neuronitis, acute unilateral vestibular hypofunction, and vestibulitis, have been used in the literature to describe the clinical syndrome presented with acute onset and severe dizziness that lasts days or weeks, which is occasionally associated with MRI enhancement of the vestibular nerve. Vestibular neuritis can affect almost any combination of afferent pathways in the vestibular nerve (48, 49).

The video head impulse test has gained great popularity as a frontline vestibular testing tool. The ease of performing vHIT during acute vertigo, especially in cases of acute vestibular syndrome, its ability to measure the VOR gain of the six semicircular canals, and its ability to detect overt, covert, and anti-compensatory saccades made it a crucial part of routine vestibular assessment.

Different commercial vHIT systems are available in the market, with a significant amount of research based on their clinical use (11). Acute unilateral vestibular hypofunction is a condition that is frequently encountered in clinical practice. VOR gain is typically reduced in one or more of the semicircular canals ipsilateral to the side of hypofunction. The literature showed that the main differential diagnosis of acute unilateral vestibular hypofunction is posterior circulation stroke specifically AICA and PICA. A VOR gain of lateral canal vHIT can differentiate between dysfunction of the vestibular end organs or their afferents and its central mimics: PICA and AICA infarctions (17).

The results of the conducted systemic review in this study revealed that the VOR gain of horizontal SCC ranged between 0.15 and 0.89 ipsilesional in cases of vestibular neuritis. On the contrary, the VOR gain of the lateral canal is usually spared or minimally reduced in PICA infarctions. Variable VOR gain values were reported in very few cases of AICA infarction as indicated in the study by Manokoudis et al. This is expected as AICA infarction could be associated with labyrinthine ischemia, leading to damage of vestibular end organs or their afferents. It could also be associated with damage to vestibular nuclei or cerebellar structures as flocculus but it sometimes spares the integrity of the lateral canal VOR. Thus, different patterns of VOR gain values could be seen in cases of AICA (17). This agrees with findings reported by Chen et al. using a magnetic search coil (50).

Among the patients with vestibular neuritis, the various studies' results suggest a greater variability in the range of vestibule-ocular reflex gain. (18, 42). One of the studies included in the review reported the range of ipsilesional VOR gain in cases of vestibular neuritis to be between 0.15 and 1.18 (mean VOR gain = 0.49 ± 0.03), and for the contralesional side to be ranged between 0.70 and 1.28 (37). This means some of the included subjects presented with spontaneous nystagmus, caloric weakness, normal lateral canal VOR gain, and no signs of posterior circulation stroke. These cases possibly do not have vestibular neuritis but rather Meniere's disease or these results may be due to faulty calibration during vHIT. In fact, this is the only study that reported occasional high VOR gain in vestibular neuritis.

Most studies suggest a reduced vestibular ocular reflex gain in patients with vestibular neuritis. The studies suggested that the most commonly affected SCC is the lateral canal, which is why this research focuses on lateral canal VOR gain because it is more clinically significant. (18, 43). Cherchi and Yacovino (49) explained why the lateral canal is the most vulnerable semicircular canal to different pathologies with the anatomical fact that it has a lower number of afferent neurons which are condensed in a limited space. Clinical protocols used to differentiate vestibular neuritis from stroke such as HINTS protocol mainly depend on lateral canal VOR gain loss and compensatory saccades (1).

In a healthy subject during an abrupt head turn to the left, receptors and afferents in the left horizontal canal are activated and *simultaneously* receptors and afferents from the right horizontal canal are inhibited. Also, increased activity in the ipsilateral vestibular nuclei leads to decreased activity in the contralateral vestibular nuclei which in turn decreases the inhibitory effect on the ipsilateral vestibular nuclei, leading to enhancement of ipsilateral activity. In this way, the functionally inhibitory commissural connections act to enhance the difference in neural activity between the two vestibular nuclei. This explains the slightly reduced contralateral VOR gain in cases of acute unilateral vestibular hypofunction as shown in different studies (11).

Based on the results of the systematic review of VOR gain values in acute unilateral vestibular hypofunction, the authors propose a clinically needed grading scale for the severity of VOR. Based on the data presented in this study, both mild and profound VOR gain loss can present as acute prolonged vertigo with spontaneous nystagmus but recovery, compensation, duration of the acute symptoms, and residual symptoms are not expected to be the same.

The proposed scale is as the following: ≥ 0.8 normal, 0.79–0.70 mild loss, 0.69–0.4 moderate loss, 0.39–0.2 severe, < 0.2 profound loss. This scale could be used similarly to the hearing loss grading scale (25) which is widely used for the adjective description of hearing loss.

### Limitations of the study

The study is limited to lateral canal VOR gain and does not include anterior and posterior semicircular canal VOR gain. The included studies used different video head impulse test systems and, consequently, different VOR gain measurement methods. The inclusion criteria of subjects with acute unilateral vestibular hypofunction are not standardized in all the included studies. The study only included acute unilateral vestibular hypofunction due to vestibular neuritis, PICA, and AICA but not due to other pathologies. Another limitation of this work is that it is focused on numerical values of VOR gain with a lack of information about the morphology of the oculomotor response and the presence of corrective saccades, which are considered clinically significant parameters of vHIT interpretation.

## Conclusions

VOR gain loss grading could provide an easy adjective description of variable degrees of lateral canal VOR gain loss due to different pathologies. Calculated VOR gain value of the lateral canal using video head impulse test helps in the etiological diagnosis of acute vestibular syndrome. The adjective scaling proposed in this study would make this much easier. For example, if the VOR gain of the ipsilesional lateral canal shows moderate to profound VOR gain loss with normal or mild VOR gain loss on the contralesional side, this is most likely a case of vestibular neuritis. If the VOR gain is normal or mildly reduced on both sides, it is most likely a PICA stroke. For an AICA stroke, there is a variety of reported patterns, and different degrees of ipsilesional and contralesional VOR gain loss could be found.

## Recommendations

Further studies applying the VOR gain loss scale in different disorders, e.g., Meniere's disease, migraine, and transient ischemic attacks, should be conducted. Further, more studies with a larger number of subjects should be conducted.

## Data availability statement

The original contributions presented in the study are included in the article, further inquiries can be directed to the corresponding authors.

## Author contributions

MAlf conceptualized the work, contributed to data collection, analysis, contributed to writing, and revising the manuscript. MAlg and SS contributed to data collection, analysis, writing, and revising the manuscript. AN contributed to data collection and the analysis of data. All authors contributed to the article and approved the submitted version.
